# Colorado sunflower water use, physiology and productivity dataset

**DOI:** 10.1016/j.dib.2025.111959

**Published:** 2025-08-06

**Authors:** Thomas J. Trout, Louise H. Comas, Kendall C. DeJonge, Huihui Zhang

**Affiliations:** Water Management and Systems Research Unit, USDA-Agricultural Research Service, 2150 Centre Dr., Bldg D, Fort Collins, CO 80526 USA

**Keywords:** Agronomy, Crop ecophysiology, Deficit irrigation, Evapotranspiration, Water stress, Root Growth, *Helianthus annuus*

## Abstract

The USDA-Agricultural Research Service conducted deficit irrigation and water productivity field trials for irrigated sunflower (*Helianthus annuus*) in northeastern Colorado from 2008 to 2016. The dataset from these field trials, composed of 10 Excel spreadsheet workbooks, is available online from the USDA National Agricultural Library “Ag Data Commons”. The dataset includes measurements of irrigation, precipitation, soil water storage, and periodic plant responses; daily estimates of crop evapotranspiration; and seasonal crop water use, biomass, and yield data. Soil parameters, hourly and daily weather data, management practices, and photo images are also provided. Each data file includes a data dictionary. The dataset can be useful to develop and evaluate sunflower crop growth and water use models under a wide range of water availability.

Specifications Table

**Table:** 

Subject	Earth & Environmental Sciences
Specific subject area	This dataset quantifies the water use and productivity of oil-type sunflower in a semi-arid region of the U.S. Central Plains. Sunflower response and productivity were measured for 9 growing seasons in a field trial under a wide range of water availability amount and timing through managed deficit irrigation. Crop water use was calculated by water balance methodologies. Crop phenological, physiological and productivity responses are documented.
Type of data	Data are biological and physical data collected in agricultural fields. Data are in both spreadsheet (.xlsx) and comma delimited (.csv) formats. Includes Raw, Analysed, Compiled, Tables, Figures, Images
Data collection	Data were collected in a randomized block field trial with 6 or 12 levels and timing of water availability and four replications for 9 growing seasons. Hourly weather parameters were measured with an on-site weather station. Irrigation amounts were carefully scheduled and measured with flow meters and applied via micro-irrigation. Soil water content was measured with a neutron moisture meter to 200 cm depth and a time domain reflectometer in the surface soil layer. Soil properties were measured in the laboratory with standard soil physical methodologies and in the field. Daily and seasonal crop water use was calculated. Agronomic practices were documented. Plant phenology, growth and structure were observed and measured by digital image analysis and intercepted photosynthetically active radiation measurement. Biomass and seed yield were measured.
Data source location	The field experiments were carried out at the USDA-ARS Limited Irrigation Research Farm (LIRF) located NE of Greeley, CO., U.S.A (40°26′ N, 104°38′ W, and 1428 m asl).
Data accessibility	Repository name: United States Department of Agriculture, National Agricultural Library, Ag Data Commons Data identification number: doi.org/10.15482/USDA.ADC/28,586,066 Direct URL to data: https://doi.org/10.15482/USDA.ADC/28,586,066
Related research article	Trout, T.J., K.C. DeJonge and H. Zhang, 2025. Crop Water Use and Crop Coefficients of Sunflower in the US Great Plains. Ag Wat Man https://doi.org/10.1016/j.agwat.2025.109583

## 1 Value of the Data

• Can be used by crop modelers to develop, calibrate, and/or validate sunflower crop models over a range of water availability conditions.
• Can be used to develop relationships between phenological, physiological, or productivity outcomes and deficit irrigation or crop water stress.
• Can be used to test and validate crop water use, evapotranspiration, and irrigation requirement models.

## 2 Background

Semi-arid agricultural regions are experiencing uncertainty and competition for limited water supplies. To maintain economic production, water productivity (production per unit water consumed) must be maximized. The study was conducted to determine the water productivity of sunflower. This dataset will be valuable to develop, parameterize, and validate sunflower crop models that can be used to generalize the results to other regions and conditions. It also can be used to validate crop water use and irrigation scheduling models. An unusual aspect of the dataset is it includes 9 years of field data with a wide range of environmental conditions and watering regimes.

## 3 Data Description

This dataset includes water use and crop response and yield measurements for sunflower (*Helianthus annuus*) for 9 cropping seasons (2008–2016). The methodology includes detailed measurement of water balance components so crop water use (evapotranspiration) can be calculated.

Data include:

• Soil texture and water retention vs. water potential
• Hourly and daily precipitation, air temperature, relative humidity, solar radiation, and wind speed
• Irrigation applications
• Soil water content
• Crop growth and phenology (growth stages, canopy ground cover, crop height, leaf area index, light interception, root growth)
• Crop physiology (effective quantum yield of chlorophyll fluorescence, midday leaf water potential, and stomatal conductance)
• Final above ground biomass, seed yield, and harvest index
• Crop management activities, including tillage, fertility, and pest control

Calculated, inferred, and estimated data include:

• Soil field capacity and total available water
• Reference evapotranspiration
• Crop evapotranspiration
• Crop transpiration
• Soil evaporation

The data are presented in spreadsheet format (.xlsx). The primary data are the ***Daily Waterbalance.xlsx*** file that includes the daily water balance and phenology data and calculated crop water use and an ***Annual Data.xlsx*** file that includes seasonal crop water used, crop biomass and yield data. Additional data files include daily and hourly weather parameters and calculated reference evapotranspiration (***LIRF Weather 2008–2016.xlsx***), periodic soil water content data (***SoilWater.xlsx***), periodic crop growth and physiology data (***Biomass.xlsx***), (***Canopy Cover.xlsx***), (***Sunflower Physiology.xlsx***) and (***Root Production 2012.xlsx***), and annual crop management logs (***Crop* Log*.xlsx***). Each spreadsheet contains a *Data Descriptions* worksheet that provides worksheet and column specific information. Notes are embedded in cells with specific metadata. Blank cells indicate data not available. The ***LIRF Soils.xlsx*** file gives detailed soil information of the experimental area and ***Photos.pdf*** provide images of the crop conditions and measurement processes. The ***LIRF Sunflower Metholology.pdf*** file details the methodology used in the study.

## 4 Experimental Design, Materials and Methods

### 4.1 Field site and conditions

The field experiments were carried out at the USDA-ARS Limited Irrigation Research Farm (LIRF) (40°26′ N, 104°38′ W, and 1428 m asl) located NE of Greeley, CO. The 16-ha facility near the western edge of the U.S. Central Great Plains was developed to conduct irrigated crop water requirements and response research. Annual and cropping season average precipitation during the 9 years of the study was 312 and 142 mm, respectively.

### 4.2 Soils

The largest portion of the 5 Ha experimental area contains Olney fine sandy loam soil (fine-loamy, mixed, superactive, mesic Ustic Haplargids). Other soils in the field are Nunn clay loam (fine, smectitic, mesic Aridic Argiustolls) (blocks 3 and 4 of section D), and Otero sandy loam (coarse-loamy, mixed, superactive, calcareous, mesic Aridic Ustorthents) (most of section A) [1].

The surface soil at the site is fairly uniform due to a land levelling operation in 1980 in which the surface 20 - 30 cm was removed, stockpiled, and replaced. Below the surface layer, soil texture of the alluvial soil varies horizontally and vertically. Field soil variability was mapped in 2007, 2008, and 2012 by measuring electrical conductivity with a Veris resistivity machine (Veris Technologies, Salina, KS). In 2007 sixteen soil cores, one from each block of each field section, were collected to 1.8 m depth. Cores were divided into depth horizons by visual texture and color changes. Soil texture was measured with a hydrometer [2]. Soil texture of most soil horizons classified as loamy sand (LS), sandy loam (SL) or sandy clay loam (SCL).

Soil bulk density was measured during annual neutron moisture meter (NMM) calibration on 10 cm long volumetric samples collected with a 33 mm diameter soil tube that was inserted into the soil with a hydraulic soil sampling machine (Giddings Machine Co., Windsor, CO). Bulk density data for 93 cores are given in the Bulk Density worksheet of the ***LIRF Soils*** workbook. Average bulk density was 1.48 in the surface 15 cm and 1.68 - 1.70 g cm^−3^ in subsurface horizons and was relatively uniform spatially and with depth below the surface layer.

Twenty-seven of the 122 soil horizon samples from the 16 core samples were selected to represent a range of soil textures to determine the soil water retention characteristics. Soil water retention at 3, 10, 33, 50, 100, and 1474 kPa pressure was measured in a pressure plate apparatus [3]. Based on these measurements, permanent wilting point, *PWP*, was determined to be 50 % of field capacity, *FC*, and thus total plant available water, *TAW* (= *FC* – *PWP*), was assumed to be 50 % of field capacity.

Soil organic matter, pH, electrical conductivity and several chemical constituents were measured by the Colorado State University Soil Testing Laboratory [4] on the 2007 soil samples used for the soil water retention measurements and on annual pre-plant soil fertility samples.

Field *SWC* measurements indicated that the variability present in the field was not adequately represented by the pressure plate data. Thus, the field capacity values used in the water balance calculations were measured in the field each season for each plot *SWC* measurement site (neutron tube location) and each measurement depth. The *FC* used in the water balance was the *SWC* measured approximately 24 h after a large irrigation or precipitation event when *SWC* increased lower in the soil profile (i.e. *FC* was exceeded and drainage occurred). Thus, this value is equivalent to a 24 hr drain down *SWC* and represents the upper limit of water stored and available for plant uptake. After 24 hrs, drainage and water uptake by the plant in the active root zone generally halted downward water movement.

### 4.3 Experimental design

**Fig. 1:**
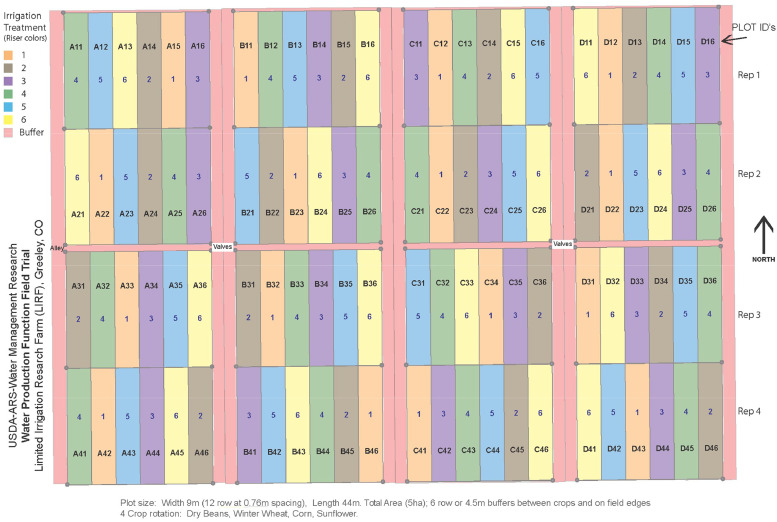
2008 – 2011 field experimental plot layout showing 4 field sections (left to right), 4 replicated blocks (top to bottom), and 6 randomly assigned treatments within each section block. Numbers in the plots refer to treatments 1 – 6. Plot identifiers refer to field section (A – D), replication block (1 – 4), and location within the block (1 – 6).

A 5 Ha field at LIRF was divided into 4 sections - one for each crop of a 4-crop rotation: maize, sunflower, pinto bean, and winter wheat (Fig. 1). Sunflower was in a different section each year following maize that was harvested the previous fall. Each section was laid out in 2008 in a randomized block design with 4 replications and 6 treatments. Each 9 × 40 m plot contained 12 N-S rows planted on 0.76 m spacing. Each section included 6 border rows on the east and west edges. All SWC and plant measurements were collected from the center 6 rows of the plots.

One of six irrigation treatments was randomly assigned to each plot in each block. The 6 treatments were designed to meet portions of full crop water requirements during non-critical growth stages. Treatments and their codes used in the dataset are:

1. 100 % of crop water requirements (no stress)
2. 85 % of treatment 1
3. 75 % of treatment 1
4. 70 % of treatment 1
5. 55 % of treatment 1
6. 40 % of treatment 1

The full irrigation treatment (tmnt code 1), was irrigated such that water availability (irrigation plus precipitation plus stored soil water) was adequate to meet crop water requirements, as predicted by the reference evapotranspiration and crop coefficients (FAO-56 methodology, [5]). Adequacy was monitored by ensuring the *SWC* remained in the plant readily available range (assumed 60 % of *TAW*). The remaining treatments were irrigated to achieve total water applications (irrigation plus precipitation) that approximated the target treatment amounts during non-critical growth stages.

All treatments were fully irrigated until growth stage V12 [6] to promote good crop stands and proper formation of reproductive organs. Stress treatments were then applied during the remaining vegetative and early reproductive growth stages (V12 – R3). Water stress was partially reduced by additional irrigation for all deficit treatments during the main reproductive growth stages (R3 – R6) to ensure adequate pollination and seed initiation. Stress treatments were re-imposed for the remainder of the season (R6 – R9).

**Fig. 2:**
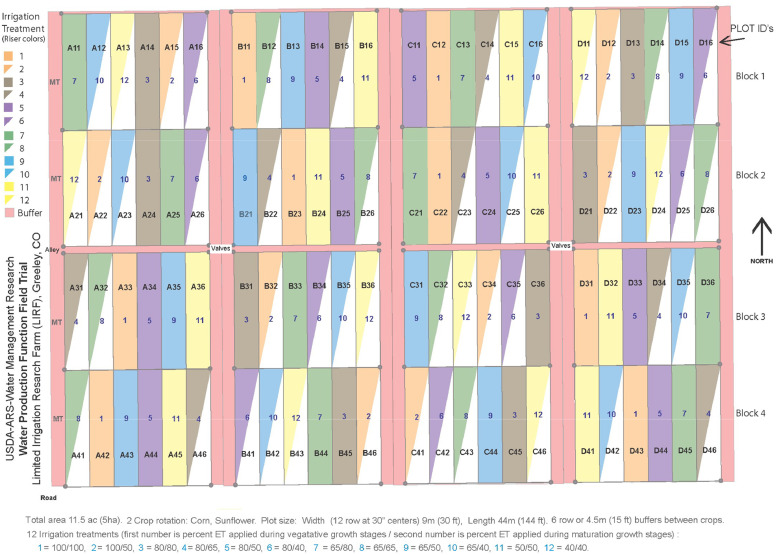
2012 – 2016 field experimental plot layout showing two combined field sections, 4 replicated blocks, and 12 randomly assigned treatments. Numbers in the plots refer to treatments 1 – 12. Plot identifiers refer to field section (A – D), replication block (1 – 4), and location within the block (1 – 12).

In 2012, deficit irrigation treatments were changed to study the impact of the timing of water stress in more detail. Section size was increased by combining two western sections (A and B) and the two eastern sections (C and D) and only two crops, maize and sunflower, were planted in each section in alternate years (Fig. 2). This allowed 12 irrigation treatments. As previously, all treatments received full irrigation up to growth stage V12, and stress was relieved through additional irrigations during the reproductive period (R3 – R6). Treatments in 2012 – 2016 were selected based on the relative deficit levels during vegetative (V9 – R3) and maturation (R6 – R9) periods as shown in Table 1.

**Table 1:** Target deficit levels for the respective vegetative and maturation growth stages for each treatment where 100 % represents full irrigation requirements.

			Maturation Stage			
	% of ET	100	80	65	50	40
	100	1			2	
Vegetative	80		3	4	5	6
Stage	65		7	8	9	10
	50				11	
	40		13			12

In 2014 – 2016, treatment 5 was discontinued and replaced with a 40/80 treatment coded as treatment 13. Under this regime, treatments 1, 3, 8, 11, and 12 were similar to 2008 – 2011 treatments 1, 2, 4, 5, and 6, respectively.

### 4.4 Crop and irrigation management

The crop was managed to achieve high yields under fully-irrigated conditions. All treatments were planted at the same population and received the same nitrogen applications. Minimum (strip) tillage was used to maintain surface residue from the previous maize crop and reduce surface evaporation. Planting dates, harvest dates, and pest management are given in the ***Crop* Log** and ***Daily Waterbalance*** workbooks.

DeKalb/Syngenta brand variety 38–45 (2008–11) or 34–95 Clearfield® (2012–16) was planted with a John Deere Maxiplex® planter in early June at 50,000-to-60,000 seeds ha^−1^. These were oil-type sunflower varieties commonly grown in the area and have similar growth and yield characteristics. Pre- or post-plant sprinkler irrigation was used when necessary to incorporate herbicide or improve germination. Final plant density averaged 3.4 plants *m*^−2^ in 2008–11 and 4.6 m^−2^ in the remaining years and did not vary significantly by treatment. Plant populations were lower in 2008–2011 than recommended for the region for irrigated oil-type sunflower (4 - 5 m^−2^) [7].

Nitrogen and phosphorus liquid fertilizer was sidedress applied near the seed at planting at 34 kg ha^−1^ N. Additional nitrogen (Urea ammonium nitrate, UAN, 32 %) was applied through the irrigation water (fertigation) to meet fertility requirements based on pre-plant soil tests, expected yields, and nitrogen concentration in the groundwater used for water supply. Note that, due to the high nitrate concentration in the groundwater (25 mg *L*^−1^ N which is equivalent to 0.25 kg N ha^−1^ mm^−1^ of water applied), low water treatments received as much as 50 kg ha^−1^ less N than the high water treatments.

Irrigation water from a groundwater well was applied through a surface drip irrigation system with drip tubing beside each row. The irrigation system was installed each season after planting and removed before harvest to avoid interference with farming operations. Although the groundwater was relatively high in soluble salts (EC = 1.9 dS *m*^−1^), there was no evidence of salinity levels in the soils that would impact yield (1:1 extraction soil water EC < 0.5 dS *m*^−1^). Irrigations were applied once or twice each week (every 4 or 5 days). Irrigation amount for the full irrigation treatment was adjusted as needed for measured soil water deficits to maintain *SWC* in the readily available range. Irrigations were reduced based on received and anticipated precipitation. Irrigation amounts for the remaining treatments were reduced relative to the full irrigation treatment to achieve the targeted crop water transpiration reductions. If required irrigation amount was <12 mm, the irrigation was skipped since there was sufficient readily available water for the crop until the following irrigation and a larger portion of small irrigation amounts is lost to surface evaporation.

A log of miscellaneous crop management operations including tillage, planting, harvest, and pest management is listed in the ***Crop* Log** workbook.

### 4.5 General crop conditions

The goal was to create vigorous crops with full yield potential. As is often the case with field experiments, these goals were not always met. Failure to achieve target uniform plant density (> 4 plants *m*^−2^) is a common problem with sunflower. The ***Crop* Log** workbook lists practices that were used to facilitate germination (sprinkler irrigation) and emergence (tillage and sprinkler irrigation to reduce the soil crusts). When plant density was less than desired due to uneven plant emergence resulting from to poor germination, soil crusting, and/or bird and mice predation of planted seeds, either plants were transplanted from border areas to sampling areas or areas were re-seeded. Partial transplanting or reseeding was done in 2009, 2013, and 2016. This resulted in improved density but non-uniform plant growth. Sunflower plants expand their canopy and head size to compensate for missing plants [7,8] and mid-season canopy ground cover, f_c_, exceeded 80 % in all years. Soil water content and biomass sampling areas were selected to represent uniform crop stand and avoid areas with low plant density.

When density and/or canopy ground cover were unacceptably low in selected plots, those plots were removed from the data treatment means. In no case were more than >1 plot from a treatment removed. The removed plots were: 2010: B16 (treatment 6); 2013: A21 (12); 2014: C31 (9), C32(8), and C33(12); 2015: A32 (8), B22 (4). In 2009 excess application of herbicide caused crop damage such that many plots did not have viable crop density. Two adjoining plots in each block were selected based on acceptable density and treatments 1 and 6 were randomly assigned to these plots while measurements on the remaining plots were terminated. Thus, in 2009, only two treatments were documented. In 2013 and 2016, plant density was low in many plots due to uneven plant emergence and growth. Although reseeding resulted in acceptable canopy cover, late emerging replants impacted crop yield non-uniformly so sunflower yield is not reported for 2013 and 2016.

Mid-summer hail is common in the Central Plains. Hail occurred on July 27, 2009; July 17, 2016, and August 19, 2016. The 2009 hail event just before R1 stage (DOY 210) damaged leaves and reduced the horizontal canopy structure. In 2016, the August hail (DOY 232, growth stage R6) greatly reduced leaf area and canopy ground cover for the remainder of the season and likely reduced end-of-season above-ground biomass.

Birds feed on mature sunflower seeds. In 2009, bird predation was substantial especially in blocks 1 and 2. Heads were sampled in each block and percent seed loss to birds was assessed as 12 – 16 % in blocks 1 and 2 and 4 – 7 % in blocks 3 and 4. Yields were adjusted for this seed loss (adjusted yield = measured yield/(1-ratio of seed loss). Bird damage was avoided in yield sampling areas in following years with various techniques including covering nets. In 2012, sunflower moth caterpillar damage resulted in stem mold and partial loss of head viability. The damage was assessed in each plot and yields were adjusted as with the earlier bird damage. The yield average adjustment was 5 %.

### 4.6 Water balance measurements

Weather parameters were measured with a standardized reference evapotranspiration weather station located on a 0.4 Ha irrigated grass plot adjacent to the experimental field. The station, part of the Colorado Agricultural Meteorological Network, station GLY04 [9], measured solar radiation, relative humidity, wind speed and direction, air temperature, and precipitation. Instrumentation is described in the ***LIRF Weather 2008 - 2016*** workbook Data Dictionary.

Both hourly and daily weather parameters are provided. The weather data were checked for errors by comparing with nearby weather stations and expected ranges. When errors were detected, or data were missing, data were replaced by measurements from a nearby weather station, as noted in the data files. Reference evapotranspiration was calculated from hourly weather data by the ASCE Standardized Penman-Monteith equation [10]. Both tall (alfalfa), *ET_r_*, and short (grass), *ET_o_*, reference evapotranspiration are presented. Daily reference values are sum of hourly values.

Two additional precipitation gauges were located inside the experimental field. Daily precipitation data are the average of the three gauges or the average of two if one gauge malfunctioned. Generally, at least 2 gauges recorded precipitation events within 1 mm. The tipping bucket gauges do not accurately measure winter snow, so winter precipitation was estimated from other gauges in the local area.

Irrigation applications to each treatment were measured with turbine flow meters (Badger Recordall Turbo 160 with RTR transmitters, Badger Meters, Milwaukie, Wisconsin). The meters were tested by the manufacturer to within ± 1.5 % accuracy. New meters were cross calibrated in the authors’ hydraulics laboratory to within ±2 % at the beginning of the study, and to within ±3 % after 8 years of use (2015). Irrigation applications were controlled by and recorded with Campbell Scientific CR1000 data loggers. Irrigation water was delivered to the corner of each plot through underground PVC pipe, to each row through a polyethylene tubing manifold, and applied to the crop from drip irrigation tubing (16 mm diameter, thick-walled tubing with 1.1 L *h*^−1^ conventional inline emitters spaced 30 cm apart) placed on the soil surface near each row. A constant pressure water supply controlled with a variable speed drive booster pump, low pressure loss in the delivery system, annual acid flushing of the drip tubing, and relatively flat topography resulted in predicted water distribution uniformity exceeding 95 %.

Soil water content, *SWC*, was measured 2 or 3 times each week before irrigation and the day after about two-thirds of irrigations in the crop row near the center of each plot. Because the water balance calculations were end-of-day, *SWC* measurements taken in the morning of an irrigation were assigned to the end of the previous day. Soil water content was measured in 30 cm depth increments between 30 and 150 cm depth, and at 200 cm depth with a neutron soil moisture meter, NMM, (CPN-503 Hydroprobe, InstroTek, San Francisco, CA). The NMM was calibrated each year at 15 % of the NMM measurement sites with volumetric soil cores collected during access tube installation. Calibration data indicated no variation in the calibration with location or depth and change with time. The calibration was used to convert instrument relative counts to volumetric *SWC*. The NMM measures *SWC* within an approximately 15 cm radius from the measurement point and was assumed to represent the soil profile within 15 cm of the measurement depth (e.g. the 30 cm depth measurement represented the 15 – 45 cm depth). The *SWC* in the surface 15 cm was measured in the row near the NMM access tube with a portable time domain reflectometer (Minitrase, Soilmoisture Equipment Corp, Santa Barbara, CA) with 15 cm long rods. Because *SWC* was measured in the crop row near the drip tubing, one-dimensional soil water models that assume water is applied uniformly across the soil surface may predict smaller fluctuations in *SWC* with time between irrigation events.

Because of variability of *FC* values among plots within a treatment, means of plot *SWC* values do not adequately represent the impact of soil water status on plant response to the treatment. Soil water deficit (*SWD* = *FC* – *SWC*) better indicates plant available water and was more uniform among plots within a treatment than *SWC*. Therefore, mean *SWD* was used to represent soil water status and used in water balance calculations, as well as determination of irrigation requirements.

In 2011, 2013, and 2015 a Bowen Ratio Energy Balance, BREB, system was used to measure well-irrigated sunflower evapotranspiration near the center of a field directly south of the experimental plots. Mkhwanazi [11] provides details of the BREB instrumentation, operation, and data analysis. The approximately 120 m x 150 m fields were planted within 2 days of the plots and were irrigated to meet full irrigation requirements. BREB evapotranspiration was calculated over 30-min intervals throughout the day and summed to obtain daily values.

### 4.7 Water balance calculations

Evapotranspiration was calculated based on the water balance (all units in equivalent depth (mm)):

(1) $$I+P=DP+RO+ET_{a}+\Delta S$$

where:

• *I* = irrigation application
• *P* = precipitation
• *DP* = deep percolation loss of soil water below the root zone
• *RO* = surface runoff of precipitation or irrigation
• *ET_a_* = actual crop evapotranspiration, the loss of water to the atmosphere
• *∆S* = change (increase) in soil water content in the root zone

Runoff due to precipitation was assumed 0 since the experimental field was fairly level, infiltration was adequate, and precipitation events were generally not large. There was no runoff from drip irrigation. Upflux from groundwater was not considered because the water table was >5 m below the surface.

Thus, for this study, *ET_a_* was calculated by water balance as:

(2) $$ET_{a}=I+P-\Delta S-DP$$

Deep percolation was assumed to occur when precipitation exceeded the *SWD* the full root zone at the time of precipitation and was calculated as the precipitation or irrigation amount minus soil water deficit. Evidence of *DP* was verified by an increase in *SWC* below the full root zone following precipitation. By these criteria, deep percolation losses occurred only in 2008 following a large multiday precipitation event. Because irrigations were carefully scheduled and applied by drip irrigation, no deep percolation loss occurred due to irrigation.

Evapotranspiration can be calculated by water balance each time *SWC* was measured (every 2 – 5 days in mid-season). However, average *ET_a_* over a multiday interval does not represent daily *ET_a_* because the atmospheric evaporative demand and thus *ET_a_* vary daily. Therefore, daily *ET_a_* was estimated by the FAO-56 method [5]:

(3) $$\begin{array}{c} ET_{a}=ET_{\mathrm{ab}}+E=K_{\mathrm{cb}}\times K_{s}\times ET_{r}+E \end{array}$$

where:

• *ET_ab_* is the basal crop evapotranspiration (*ETa* minus wet soil evaporation),
• *E* is the wet soil evaporation,
• *K_cb_* is the basal crop coefficient,
• *K_s_* is a water stress coefficient, and
• *ET*_r_ is the tall crop (alfalfa reference) evapotranspiration.

*K_cb_* was initially estimated as a linear function of the crop canopy ground cover [12]:

(4) $$\begin{array}{c} K_{\mathrm{cb}}=K_{\mathrm{cb}-\mathrm{ini}}+\left(\left(K_{\mathrm{cb}-\mathrm{mid}}-K_{\mathrm{cb}-\mathrm{ini}}\right)/0.8\right)\times f_{c} \end{array}$$

where:

• *K_cb-ini_* = *K*_*cb*_ with bare soil,
• *K_cb-mid_* = *K*_*cb*_ value at mid-season when *f_c_* ≥ 0.8, and
• *f_c_* = crop canopy ground cover with maximum value of 0.8.

*K_cb-ini_* and *K_cb-mid_* were initially estimated as 0.12 and 1.0, respectively based on typical alfalfa reference values in Jensen and Allen [13] and the upper limit for *f_c_* was 0.8. The water stress coefficient was estimated by FAO-56 procedures based on the *SWD* with readily available water = 60 % of *TAW*.

Evapotranspiration, calculated by Eq. (3) with *K_cb_* calculated by Eq. (4), was substituted into Eq. (2) solved for *∆S* to calculate daily *SWD*.

(5) $$SWD_{i}=SWD_{i-1}-\Delta S=SWD_{i-1}-I-P+DP+ET_{a}$$

where:

• *SWD_i_* = current day *SWD*
• SWD_i-1_ = previous day *SWD*

When the model-predicted *SWD* trend deviated from measured *SWD, K_cb_* was manually adjusted in small increments for a preceding time period so that the predicted *SWD* trend matched the measured *SWD* data. This process was used to interpolate daily *SWD*. The resulting daily *ET_a_* values were thus “calibrated” to match the long-term water balance measured *ET_a_*, and the sum of these daily values was equal to the cumulative *ET_a_* predicted by Eq (2) over multi-week and seasonal time intervals. Although the daily *ET*_*a*_ values were calibration estimates, seasonal *ET_a_* values were based directly on the measured water balance parameters.

The transpiration component of *ET_a_, ET_ab_*, is *ET_a_* minus wet surface evaporation, *E*. Surface evaporation was estimated by assuming that the total evaporable water, *TEW* (assumed 12 mm), or the depth of irrigation or precipitation if less than *TEW*, evaporates from the wetted sunlit portion of the soil surface between each wetting event [5]. The entire surface is wetted by precipitation events, but only the portion of the soil near the drip tube emitters is wetted during irrigation. The sunlit soil is reduced by the crop canopy ground cover and residue on the surface. Since the drip tube is near the plant row, the plant canopy shades much of the irrigation wetted surface for much of the season. Thus, this model will predict less surface evaporation from irrigation than one-dimensional models that assume the whole soil surface is wetted. For our reduced tillage condition, residue was assumed to shade 20 % of the surface. When the surface soil was wet, we assumed daily *ET_a_* was limited to 105 % of *ET_r_*, so daily soil evaporation was limited to *E* ≤ 1.05 – (*K_cb_* × *K_s_*) × *ET_r_*.

The daily *K_cb_* values in the dataset were both the calibrated values described above and values calculated directly from the waterbalance. Because small inaccuracies in *SWC* measurements or differences in *SWC* measurement times result in relatively larger errors in ∆S over short time intervals in which cumulative *ET_a_* is small, this calculation resulted in relatively large variability over time in calculated *K_cb_*. Therefore the presented waterbalance daily *ET_a_* values are 15-day moving averages of the measured *ET_a_* values. This interval was long enough to include 2 or 3 WB measurements but short enough to follow general *K_cb_* trends over time.

Effective rooting depth was estimated based on observed soil water uptake (measured *SWC* decrease). No soil water uptake was measured below 1350 mm depth, so the final root zone depth for sunflower in the experimental field was estimated to be 1350 mm (represented by the 0 – 15, 30, 60, 90, and 120 cm *SWC* measurements). The root zone depth during the growing season was modeled by a cubic spline between plant emergence (100 mm) and full cover (1350 mm).

### 4.8 Plant measurements

Plant density was measured before harvest by counting the total number of plants in all or a portion of the plot yield sample area (described below). Plant density was generally less than the seeding rate (5 – 6 seeds *m*^−2^) due to seeds that failed to germinate and become viable plants. Non-consecutive plant skips do not substantially reduce evapotranspiration or yield because neighboring plants and sunflower heads expand to fill available space [7].

Growth stage was periodically evaluated visually based on guidelines presented in Schneiter and Miller [6]. Plant height was measured at maximum height in 2008, 2010, 2011, 2012, 2013, and 2014) and in 2010 – 2012 during plant growth, with a measurement rod from the soil surface to the top of the leaf canopy or sunflower head.

Crop canopy ground cover was measured approximately weekly near solar noon with a digital camera from a nadir view six meters above the ground surface. The camera field of view encompassed 4 rows x 4 m. The digital image pixels were differentiated between green plant canopy and background (soil, surface residue, and senesced leaves) with manually-trained image analysis software. Since canopy ground cover growth and senescence are continuous processes, daily values were interpolated by a least squares polynomial fit to the data. Canopy ground cover in stressed sunflower may be reduced by leaf droop that occurs under high evaporative demand primarily in the afternoon. Since the intent of the measurement was to measure the impact of crop canopy on evapotranspiration, measurements below trends were assumed to result from leaf droop were not included in the interpolation.

Intercepted photosynthetically active radiation (IPAR) was measured within 30 min of solar noon with a light bar (Sunfleck or AccuPAR LP-80; Decagon Devices, Pullman, Washington, USA). Measurements were taken at five locations in each plot (same location in each plot over the season), from the base of a plant in one row to the base of a plant in the adjacent row.

Above ground biomass was sampled near the end of the season (after physiological maturity) each year plus 3 earlier dates during the season in 2012, one earlier date in 2014, and two earlier dates in 2015. Five adjoining plants in a row were sampled in two locations in each plot. Sample sites were selected in row sections with high plant density (few if any missing plants). Plants were divided into stalks, leaves, and heads, and dried at 60° C for 48 h and weighed. Seeds (kernel and hull) were then removed from the heads and weighed. Average total above ground biomass and seed yield per plant were multiplied by the plant density to derive values per unit area (kg ha^−1^). In 2013 – 2016, biomass plant density was based on the biomass sample area. In 2008 - 2012, biomass sample area was not measured so plant density was based on average plant density of the plot which was generally lower than the biomass sample area density due to occasional missing plants. Because sunflower plants and heads tend to be larger when density is low, multiplying per plant biomass by plot density likely underestimates plot biomass. Harvest Index was calculated as the ratio of the dried weight of seed to the dried total above ground biomass. Due to the small area of the biomass sample, the biomass seed yield is considered less accurate than the larger plot harvest yield measurement.

Leaf Area Index (LAI) was measured on a subset of biomass samples in several years. Leaves were cut from stems and scanned with a leaf area meter (LI-3100C; LI-COR, Lincoln, Nebraska, USA), and then leaves from each sample were dried and weighed to determine the ratio of fresh leaf area to leaf dry mass (specific leaf area; SLA, cm^2^ g^−1^). If SLA did not differ statistically among treatments, the mean SLA across all treatments was used to calculate leaf areas for that sampling time, otherwise the SLA of the most similar irrigation target was used. LAI was calculated as the SLA multiplied by the dry leaf mass per plant times the plant density.

Sunflower yield was measured at the end of the season after seeds had dried to approximately 10 % moisture content by hand harvesting the sunflower heads from the middle section (15-to-20 m) of the center 4 rows of each plot. Sunflower seeds (cypsela = kernel plus hull) were removed from the heads with a stationary thresher (Wintersteiger Classic ST, Wintersteiger AG, Ried, Austria), and weighed. Seed water content at harvest was measured on a yield subsample with a Dickey-john GAC500-XT Moisture Tester (Dickey-john Corp, Auburn, Ill). Yield (kg ha^−1^) was normalized to 10 % moisture content (commercial yield standard). Thus, dry seed weight (0 % moisture) is 90 % of reported seed weight. In 2009, 2012, 2013, and 2014, a subsample of seeds was dried (60 °C for 48 hrs) and the mass of 200 seeds was measured and converted to mass per 1000 seeds. In 2008, 2010, 2012, and 2013, seed oil content was measured (percent of kernel weight at 10 % kernel moisture content) by the USDA-ARS Sunflower Improvement Research Unit, Fargo, N.D.

Measurements of sunflower effective quantum yield (light-adapted chlorophyll fluorescence, Fm' - Fs)/Fm'), midday leaf water potential (*LWP*), and stomatal conductance (gs) were measured in 2012. Effective quantum yield was measured as a pulse amplitude modulated signal on one of the top 2 fully expanded leaves that were fully sunlit. Measurements were taken with a fluorometer (OS5p; Opti-Sciences Inc, Hudson, New Hampshire, USA) halfway between the tip and the petiole as far in from the edge as the fluorometer could reach, in a two-hour window prior to solar noon. Intensity and gain settings were optimized to ensure a measurable signal with stable plateau following a saturating flash. Midday leaf water potential (*ΨL*) was measured with a Scholander-type pressure chamber (Model 3005 Series Plant Water Status Console with an 18 cm long chamber, Soil Moisture Equipment Corp., Santa Barbara, CA, USA) within two hours past solar noon. Fully expanded leaves in the sun were cut, put in a sealed plastic bag in a cooler until measurements were made. Leaves were wrapped in a moistened cloth with the petiole fed through the lid of the chamber and re-cut immediately before pressurizing the chamber for measurements.

Root populations [14] were monitored in 2012 through transparent plastic (cellulose acetate butyrate) root observation tubes (minirhizotrons), 183 cm long and 5.7 cm outside diameter. Tubes were installed within one week of plant emergence 5–10 cm from the plant row halfway between two plants angled 30° from vertical toward the base of the nearest plant. Images of roots visible in a column of 127 windows that were 18 mm × 13.5 mm were collected every two weeks through the growing season with a miniature video camera system (BTC-2 with I-CAP image capture; Bartz Technology Corp, Carpinteria, CA, USA). Dates that individual roots were produced along with their lengths in every 5th window were recorded with RootFly 2.0 software (Clemson University, Clemson, SC, USA).

## Limitations

These data were collected under agricultural field conditions. Variations due to weather conditions and pest infestations occurred. These limitations are described above under General Crop Conditions and in the dataset.

## Ethics Statement

The authors have read and follow the ethical requirements for publication in Data in Brief and the current work does not involve human subjects, animal experiments, or any data collected from social media platforms.

## Data Availability

USDA National Ag Library, Ag Data Commons: https://agdatacommons.nal.usda.gov/USDA-ARS Colorado Sunflower Water Use and Productivity Dataset (Original data).
